# Virchow’s Pathological Views

**Published:** 1861-08

**Authors:** R. K. Browne

**Affiliations:** Professor of Physiology in the New York Medical College


### VIRCHOW'S PATHOLOGICAL VIEWS.

BY DR. R. K. BROWNE,

*Professor of Physiology in the New York Medical College.*

[Read before the New York Medico-Chirurgical College, June 27th, 1861.]

Virchow is an epoch-making author, and is justly entitled to the prefix great. His pathological doctrines mark the opening of an era during which they will lead the advance in that field of science. The existence of Virchow's "Cellular Pathologie" is well known, yet apparently but few of the medical world have examined into the purport of its contents. It consists of twenty lectures, which, in 1858, at Berlin, first came from the lips of Virchow. They are reported to have been very convincing to his auditors.

We are not about to present a summary of this volume, but merely a succinct exposition of certain of its portions. It contains but very little matter that, prior to the lectures, had not been presented by Virchow in other publications, and the student of medical science, *au courant* with the contributions made to its literature in Germany for the most recent ten years, will find in the "Cellular Pathologie" but little entirely new or surprising; and in that literature must a fuller presentation of Virchow's researches and discoveries be sought for.* "Cellular Patholigie" imports a pathology in contradistinction to the humoral and solidistic pathology, from both of which it is sufficiently distinct, without, however, involving their entire rejection.

*Cell Development.*—The leading position of Virchow, contrary to the persuasion of the older pathologists, is in his understanding of the mode of cell-production and transformations. Starting from a denial of *free* cell-production, his explanation of the mode of formation of pathological tissues is directly at variance with the general view, which is, that free cell-production constitutes the mode of growth of all pathological formations.

The same view was also held undoubtingly with regard to the physiology of normal tissue-growth, until Kolliker made the covert suggestion "that it had been much too readily taken for granted. (*Microscopical Anat.*)

A brief sketch of the essential points of the doctrine of cell-production as they appeared from time to time in its history, rejected by Virchow, will enable those whose studies have not familiarized them with it, to understand Virchow's views of cellular growth, and his use of them in the field of pathology.

Raspail, in 1844, was the first to describe the morphology of the cell, and to give an account of the process of its formation, which he likened to crystallization. He described them as arising in "a liquid tending to organize." Raspail ascribed, therefore to the cell only an active capacity, while he ascribed to the fluid implied a formative or generative capacity, by which from itself the cells were formed. The latter idea became that of Schleiden and Schwann. But Raspail had done little more than theorize upon the observations which had accumulated in the era preceding him. We owe to Schleiden, the botanist, the first series of observations on which reposed the theory of cell-development.

He studied the process of cell-development as presented in the embryonic stage of vegetable growth. His first observations were upon the capsule which contained the albuminoid substance of the seed, and also the extremity of the pollen-tube, from which the embryonal growth arises. He repre-

* Virchow himself informs us of this; our acquaintance with his researches is recent.

sented that in both these points a solution exists which first assumes a turbid and then a granular aspect; nucleodi arise from the union of these granules at various points; next around these are found nuclei, (cytoblasts,) and subsequently the cells.

According to this account, the mode of production of the cells is the same as that of Raspail, though Schleiden spoke of its being "the formation of cells within cells;" yet, although the process took place within the large cell or embryonal sac, it is plain that each cell arose separately out of the fluid implied, and that no one of them took part in the reproduction of others. This process is what we now know as free cell-development, and this is the doctrine which Virchow rejects. Schwann followed Schleiden, and made his observations on the embryonic animal normal tissues. He had adopted Schleiden's theory of cell-production. In his examination of animal tissues, Schwann observed in the embryo innumerable cells reposing in a more or less consistent intercellular substance, and towards the production of these he entertained the idea previously announced by Schleiden. He thus assigned to the production of the cellular elements of animal tissues the doctrine Schleiden had alleged with reference to vegetable tissues. In his account of the processes subsequent to the origin of the cells, by which these are transformed into the structure, Schwann was entirely original.

This doctrine of the mode of cell origin in normal tissue was adopted by Johannes Muller in his classification of pathological formations and morbid growths. Muller led in showing that it is under the same law both normal and abnormal growths are developed; and relying upon Schwann's dictum respecting the origin of the cells, his own observations were with reference to the processes of their transformation into abnormal structure, or into degenerated morbid products.

The doctrines of Muller in pathology became those of Vogel, Lebert, Rokitansky, Paget and others. None of these reinvestigated Schwann's and Schleiden's doctries of cell-production, and their researches were limited to the investigation of the various stages of the transformation, by which morbid growths are formed out of cells after these had orginated from that blastema. But while the train of pathologists were accepting Schleiden unquestioned, and had accumulated their stores of observations in accordance with the erroneous presumption of free cell-production, the botanists, dealing with normal vegetable tissue, were diligently reinvestigating cell-growth, and in 1844 Hugo Von Mohl brought forward his explanation of the formation of cells in the plant. He affirmed that the production of cells in plants occurred only in the cavities of other cells, (plant-cells.) He differed also materially in his account of the order of the formation of the cell, its nuclei and nucleolus, from that of Schleiden; "not the nucleolus appears first, he says, "but the nuclebs or cytoblast." Von Mohl was soon followed by the investigators in animal tissues, who objected to other points of Schwann's teachings. At length, in 1852, Remak unreservedly rejected Schwann's doctrine of free cell-development, and announced the aphorism—"*Omnis cellula in cellula*"—which became the leading doctrine of Virchow, and the pedestal of the "Cellular Pathologie."

"*Omnis cellula e cellula*."*

Virchow says, "Just as little as we can now admit that teniæ can arise out of saburral mucus, or that out of the residue of the decomposition of animal or vegetable matter an infusorial animalcula, a fungus or an alga can be formed, equally little are we disposed to concede in physiological or pathological histology, that a new cell can build itself up out of any non-cellular substance. Where a cell arises, there a cell must have previously existed, (*omnis cellula e cellula*,) just as an animal can spring only from an animal, a plant from a plant."

This doctrine supplants that of Schawnn; and although Virchow was not the first observer to propound it, yet he has brought to its illustration such a wealth of observation, and has applied it in such sweeping elucidation of the normal tissue and pathological formations, especially the latter, that he is fairly entitled to the credit of being its ablest expositor.

It will be evident that without vacating the theory of free cell-production from a formative substance, plastic lymph or cyto blastema, no radical or important change of our view of morbid products or pathological formations could have been attained to. The true production of the cells in the first instance, aud the manner of the multiplication, and various transformations constituting the growth ot the tissues, was the most indispensable part of a correct understanding of either normal or pathological tissue development, and Virchow believes with Johannus Muller, that the law is similar for the growth of both these. Until the day of Virchow, the path-

* Virchow, Beit. Zur. Spec. Pathologie and Therapie., 1854.

ologists had supposed the various pathological or morbid developments to be in the first instance a blastema or plasma, exuded from the vessels of the diseased parts, and that this itself was developed by its own capacity into the cellular. growth which constituted the new formations. The degeneration of the histological forms constituted the final stage of the morbid products, as pus, etc. It is the doctrine of blastema and cell-growth that Virchow displaces by substituting the histological elements, we shall now describe.

Says Kolliker, "free cell-formation is exceedingly frequent in pathological productions, and the cells in pus and exudations of all kinds arise in this manner; in fact, all pathological cell-formation properly comes under this head." (*Microscopical Anatomy.*)

According to Virchow, the great majority of all new pathological formations arise from the excessive and perverted growth of the corpuscles of the connective tissue. Each of these, individually, consists of the cell, its nuclei and nucleoli; the constructive process consisting in all growths, malignant or benign, of the division of their nuclei and their multiplication. Instead of new formations by free cell-development from plastic lymph or blastema of Schwann, or the exudation of the early pathologists, Virchow shows that these growths are constituted of the development of the cellular element of connective tissue; the "connective tissue corpuscle" therein being the active agent of the growth, and the connective tissue itself being the seat of formation. Virchow has shown that this connective tissue, with its cellular element, the "connecnective tissue corpuscle," abounds everywhere in the human body, and that it may be regarded as enmeshing all other histological elements. Its different varieties abound profusely in the brain, liver, kidney, muscles, cartilage, and even the skin and bone; and that whatever morphological changes its cellular elements undergo, they constitute the growth of pathological formations, which are but a development of the cells of connective tissue, or a degeneration subsequently of its elements.

The cellular element of connective tissue, "the plasmatic cell," is Virchow's own discovery, and he gives the first exposition of its nature and pathological relations.

Virchow affirms that all, or nearly all, the morbid formations arrise within the connective tissue by the perverted growth of these "plasmatic cells," and makes it evident how, by the abundance of this tissue everywhere in the body, pathological formations of the same character arise in different organs, since the same producing cellular elements are equally present in the most widely separated organs.

But this being so, what is Virchow's view of connective tissue? for it is manifest that with our idea of this tissue, we cannot concede to it the functions he ascribes to it.

The histogenesis of connective tissue turns out to be related in the closest possible manner with pathological doctrine, and although recently other investigators had come to suspect that we must look to it for the growth of pathological products; yet, before Virchow, connective tissue was regarded as the most inoperative structure in the body, having none but passive properties. It was said to consist of white and yellow elastic fibres in close combination, with pigment and fat cells.

According to Schwann, the fibres of connective tissue are transformed from cells of the simplest form, which commence the process by assuming an elongated shape, thus fasten end to end, and gradually break up into fibres within *the body of the cell*, then splitting into distinct fibrils, so that each row of cells, thus mutually attached by their extremities, is developed into a bundle of connective tissue. The white fibrous bundles are sometimes described as non-cellular fibres, in very intimate conjunction with a homogeneous intercellular material, and sometimes as this very material, disposed in such a way as to present a fibrillated appearance, though not capable of separation into distinct fibrils. Notwithstanding connective tissue from the date of Schwann's researches has been the object of much investigation, Virchow was the first to give a complete and satisfactory account of its mode of formation and the character and history of its elements.

According to Virchow, the connective tissue consists of (1) white bundles; (2) elastic fibres; (3) the "connective tissue corpuscles" or cells and an intercellular substance, the same which Schwann reputed to be the blastema from which the cells were generated. The white fibres arise from the direct fibrillization of the intercellular substance, and while this is taking place some of the cells are transformed into elastic fibres, and others change to fat and pigment cells, recognized by histologists, while others of a spindle-shape, or more or less stellate, remain as a part of the tissue itself. The elastic fibres result from a peculiar change and sclerosis (hardening) of the walls of the cells, which terminate in a disappearance of the cell contents and the obliteration of their cavity. The corpuscles, sometimes fusiform, but more frequently starshaped, and joined by means of their branching prolongations, so as to form a net-work similar to that of the structure of bone, the corpuscles and and canaliculi of which Virchow asserts are really cellular, consisting of these very elements greatly transformed. As before said, while some of the embryonic cells are assuming their stellate, frequently anastamosing form and others becoming elastic fibres, the homogeneous intercellular substance becomes fibrillated, and the whole series constitute the connective tissue. By their capacity of division and multiplication, the "plasmatic cells" produce all pathological growths, whether malignant or benignant; the only distinction there is between these two being established by the kind of irritation or excitation in the part, to be explained further on. Instead, therefore, of finding the origin of new formations in plastic lymph, blastema, or organizable exudation from the blood-vessels and free cell-growth within it, Virchow substitutes for the plastic lymph, the blastema of the earlier and the plasmatic exudation of later authors, connective tissue, and especially its cellular element—"and directly traces to it the development of new formations, except epithelial formations and a few others." Virchow's doctrine, therefore, of the origin of pathological growths turns upon the fact that these cells have the function *or power of multiplying by division and originating broods of secondary cells*, in virtue of the irritation their subsequent action *supposes*, and that the developments of these cellular elements, with their outgrowths, or final degeneration, as in corpuscular lymph, pus, tubercle, constitute the vast majority of pathological formations, which are therefore growths of connective tissue. But it is not alone the cellular element of the connective tissue, but other parts of the tissue—the structural development of the cells—also undergo changes, degeneration, &c., &c.

But if the cellular elements of connective tissue are the active constituents of pathological processes, what is the inducing or exciting physiological cause of these processes? This, of course, is only to be sought in some relationship of these to external influences, and is found in the phenomena of irritability, induced when some cause of irritation acts either directly or indirectly through the medium of the blood upon the part, the normal state of which is thereby so altered, that it absorbs and attracts to itself from the parts around it a larger amount of matter than ordinarily.

*Irritability.*—Irritability pertains to the phenomenal history of every living thing, and "it is," Virchow says, "the very criterion of life." "Vital activity is, in so far as we are able to judge, nowhere, in no part whatever, carried on by means allotted to it in the beginning, but we everywhere see that a certain excitation is necessary for its production;" and accordingly, whenever a particular action is called into play, we have to deal with a manifestation of the function, the nutrition, or the formation of any part.

Irritability, therefore, is distinguished by Virchow into functional, nutritive and formative, and either of these may alone exist, or they may coexist. The action of the elements of a part may, therefore, be displayed either in the promotion of its function, its nutrition, or its formation.

The first is displayed in the performance of the normal functions; a muscle contracts, or a gland secretes, although this may be excessive, and is then considered morbid. It is to be noticed that the influences which induce the functions are various, and are not indifferent. They operate under a law of specific relation or affinities between them and the elements of the parts.

Nutritive irritability is of much more interest to the pathologist than functional. It is manifest when the nutritive activity of parts is augmented, and is defined by the conditions in which there is an increase in the size of the cells of the affected part, but no *new formation* of the cellular or other histological elements. Thus, in Bright's disease, the renal peithelial cells enlarge merely by excessive nutritive absorption. Their contents become turbid, and the tubules broadened. "If we draw a thread through a cartilage so that merely a traumatic irritation is produced, we see that all which lies close to the thread becomes enlarged through an increased absorption of material."—P. 298. This turbidity of the cells of the part and increase of their dimensions constitute a form of corneal opacity.

Finally, formative irritability is exhibited in a marked multiplication of the constitutive elements. It follows nutritive irritability. In it, we find that the cellular elements, shortly after they have experienced the nutritive enlargement, exhibit further changes, which begin in the interior of the nuclei. The nucleoli become remarkably large, sometimes oblong, and sometimes staff-shaped. Then the nucleoli become constricted in the middle and assume the form of a finger-biscuit-like constrictions, and afterwards the real division of the nucleus, also take place. This may be repeated, so that three or four nuclei arise. If now we advance a step further in these process, we come to the new formations of the cells themselves."—P. 310.

Groups of many cells arise by the continuation of this process from single ones; and in this mode of cell-production, and of these groups, consists morbid tissue growth. Their subsequent transformations and degeneration constitute the final form of the pathological product. Coincident with either of these forms of irritation may exist vascular or nervous disturbance, but they are never the inducing cause of them; "for," says Virchow, "all these forms of irritability may appear in parts not known to possess either nervous or vascular elements."

*Inflammation.*—In inflammation we have all three forms of irritation. In traumatic injury of muscle, for instance, we have nutritive and functional disturbance; and in inflammation of interstitial connective tissue, we have new formations and degenerations, pus, etc.

*Inflammation divided.*—There are two distinct forms of inflammation, but to Virchow there is no such thing as inflammatory exudation. The morbid product arising in the inflamed part is composed of material which has been generated there from the materials already there, and not deposited there from a separate source.

First, there is a purely *parenchymatous inflammation*, within the tissue, and presenting no free fluid. The cellular elements of the tissue are increased in dimensions and filled with abnormal matter. And, second, there is a *secretory* (*or exudative*) *inflammation*, belonging mostly to the mucous and serous surface. In these, the abnormal cellular product forms on the surface, attended with an increased amount of fluid.

That he has correctly conceived these cases, Virchow says, is shown by the fact that they occur in different organs. The *secretory inflammation* effects the formation of fibrin upon surfaces or in cavities, or the production of masses of mucus upon mucous surfaces; for just as the mucus is the product of the membrane carried out upon its free surface by means of the saline and watery matters of the blood, so also the fibrin, (or coagulable lymph,) instead of being a transudation from the blood-vessels, the work of vascular action, is a local product of those tissues on, and in, which it is found, and reaches the surface in the same way as the mucus of the mucous membrane.—P. 392. Absorption of the fibrin thus produced into the blood causes the increased quantity of fibrin there in inflammatory fever.

"The fibrinous crasis, therefore, is the product of local disease."

Instead of the cellular elements, *i. e.*, pus or mucous corpuscles, arising from the blastemas exuded, they are produced by the plasmatic cells, or areola tissue corpuscle of the seat of the inflammation.

"*Parenchymatous inflammation.*"—In parenchymatous inflammation, all the fluid transuded from the blood-vessels is absorbed by the cells of the parts involved, although the production of new elements may occur, or various degenerative changes may occur, (as fatty degeneration.)

The commencement of inflammation, therefore, is not in a peculiar action of the vascular or nervous tissue, but in that of the cellular elements of the part involved; and the hyperæmic condition of the vessels is not the cause of the local inflammation, but a consequence of the abnormal growth of the irritated elements of the tissues. Stop, or reduce the supply of blood, and you cut off the food which feeds their growth. "If," says Virchow, "we cut off or diminish the supply of nutritive matter, we must, of course, prevent the part from absorbing more than its wont."

*Formation of Pus.*—Virchow distinguishes two modes of pus-formation, according as pus proceeds from epithelial or from connective tissue.

If from the former, the pus-cells are produced by the multiplication of the cells of the younger strata of the epithelium, (*e. g.* the rete Malpighi of the skin.)

Says Virchow: "The more deeply-seated pus formations regularly take place in the *connective tissue*. In it, there first occurs an enlargement of the cells, (connective tissue corpuscle,) the nuclei divide, and for some time multiply excessively. This first stage is then very soon followed by divisions of the cells themselves. Round about the irritated parts, where before single cells lay, pairs or groups of cells are subsequently found, out of which a new formation of an homologous kind (connective tissue) usually constructs itself. More in the interior, on the contrary, where the cells were early abundantly filled with nuclei, heaps of little cells soon appear, which at first still preserve the direction and form of the previous connective tissue corpuscle. Somewhat later, we here find roundish collections, or diffuse infiltrations, in which the intermediate tissue is extremely scanty, and continually liquefies more and more in proportion as the proliferation of the cell extends.* After the abscess produced by such a process

*Cell. Pathologie, p. 451.

discharges, it is healed by granulations, for the formation of which it is essential that the intercellular substance should not be entirely liquefied. The difference between the suppuration and the granulation is, that in the former, the degenerative process exceeds the formative; while the reverse is the case in granulation. In the former, the intercellular substance, which is the nidus of the cells, liquefies and deprives them of further capacity of multiplication. It is an error, therefore, to attribute ulceration to the corrosive properties of pus.

"Pus is not the dissolving, but the dissolved, *i. e.*, the transformed tissue." The old structures heal up and disappear in the process of producing the new ones. The product of the cell multiplication takes the place of the previous tissue, and the ulcer will continue to increase in its size so long as this excessive cell-proliferation continues. The newly produced elements arise from prior ones which cause their structural character in the very act. So by a similar process in the production of morbid growths, the elements of the new growth arise out of the connective tissue corpuscle of the diseased tissue. Hence Virchow decides that "*there is a stage* when it is impossible to ascertain indubitably whether we have in a diseased part to deal with simple process of growth, or with the development of a heteroplastic form."—P. 454. For "the first development of cancer, of cancroid, and of sarcoma, exhibits the same stages; if the course of their development be traced sufficiently far back, we at last come across a stage in which, in the younger and deeper layers, indifferent cells are met with, which do not until a later period, according to the particular nature of the irritation to which they are exposed, assume the one or the other type."—P. 454.

In this view it is plain how microscopists have differed in the diagnosis of cancer.

Virchow classifies the new formation as heterogeneous and homologous; both, though they differ, are equally abnormal.

"Heterologous we may call not only malignant, degenerative neoplasms, but we may also thus designate every tissue which deviates from the recognized type of the part; whilst we should call all that homologous which, though new formed, still reproduces the type of its parent soil. Thus, the cells of pus are heterologous, because they differ from the normal parent cells by the proliferation of which they were produced; but tissues may be heterologous though they do not vary from physiological types.

"There is," says Virchow, "no other kind of heterology in morbid structures than the abnormal manner in which they arise, and that this abnormity consists either in the production of a structure at a point where it has no business, or at a time when it ought not to be produced, or to an extent which is at variance with the typical formation of the body."

Heterology, we are warned, must not be identified with malignity.

*Malignity.*—Cancer is malignant, not "because it contains heterologous cells, nor cancroid benignant because its cells are homologous; they are both malignant, and their malignity differs only in degree." For "(enchondromata) which were formerly described as unquestionably benignant, sometimes occur in soft, and rather gelatinous forms, which may occasion just such internal metastases as cancer."—P. 486. And sarcomata may "appear secondarily in the lymphatic glands, and in many cases occur throughout the whole body, metastatically, to much an extent that scarcely any organ is spared by them."—P. 487.

Morbid growths may infect other organs than where they arise in either of two ways. On the one hand, their cellular elements may pass into the blood, which rarely happens; or on the other, vitiated fluids of the part first affected may pass into the venous or lymphatic system, and reach some part where they may excite in the same action as occurred in their production.

A special blood disease giving rise to tumors is not admitted; but particular substances, it is admitted, may find their way into the blood, and induce particular morbid changes in individual parts by their being taken into them in virtue of a specific attraction between these parts and substances.

Very interesting to the surgical pathologist will be his account of the recurrence of certain morbific growths after extirpation.

"If we examine any proliferating tumor, of a cellular character, we often find from three to five lines beyond its apparent limits the tissues already in a state of disease, and exhibiting the first traces of a new zone. This is the chief source of local recurrence after extirpation, for it proceeds from the zone, that cannot be detected with the naked eye, beginning to grow in consequence of the increased supply of nutritive material, which results from the removal of the original tumor. No new deposit from the blood takes place there, but the new-formed germs, which already lie in the neighboring tissue, run through their further development in the same manner that it would otherwise have taken place, or perhaps still more quickly."—P. 458.

But notwithstanding Virchow rejects the ordinary distinction of malignant and benignant tumors, and affirms the similarity of their source and of the character of their histological elements and their various transformations, yet he affirms that sooner or later there are differences, that the microscope can readily be applied to detect. Accordingly, he proposes that their classification be based upon their microscopic anatomy.

His views of the genesis of tubercle are peculiarly original. What we know as the miliary form alone is truly tubercular, with which, however, he classes the bodies found in the arachnoid, and which have been denied a tubercular character, because they contain cells.

Regarding these he says:

"You will generally find the tubercle in the brain described as being solitary, but they are not simple bodies; every such mass (tuber) which is as large as a small apple, (1 in.,) or even not larger than a currant, contains many thousand of tubercles. It is quite a nest of them, which enlarges not by the growth of the original form, but rather by the continual formation and adjunction of new foci at its circumference.

"If we examine one of these perfectly yellowish-white, dry cheesy tubera, we find immediately surrounding it a soft vascular layer, which marks it off from the adjoining cerebral substance, a closely-investing areola of connective tissue and vessels. In this layer lie the small young granules, ('miliary tubercles,') now in greater, now less number. They establish themselves externally, and the large tuber grows by the continual apposition of new granules, of which every one singly becomes cheesy."

But tubercle has its origin or growth in the cells of connective tissue; it is formed of a mass of cells rich in nuclei, by a process of cell-proliferation, similar to that for pus. It is distinguished from the latter, and also from the more highly organized forms of cancer, which are formed of large corpuscles, with highly developed nuclei and nucleoli. Tubercle is also developed out of the histological elements of connective tissue, by a process of degenerative cell-proliferation. By the profusion of its growth the nutritive supply is cut off, and then it shrinks and assumes its disintegrated cheesy state. But pus or cancer may undergo a similar metamorphosis, contrary to

Sebert, who believes the corpuscles found in the centre of tubercle "to be the first bungling products, unfortunate essays of organization." Virchow affirms that they are formed out of matured elements, by shriveling and fatty degeneration. When a large tubercle corpuscle is found, "a cell had previously existed," and similarly when they occur in cancer, which similarily undergo cheesy metamorphosis; they represent the degenerated form of cancer-cells and nuclei; and so pus also undergoes cheesy metamorphosis, and its corpuscles are shriveled pus-cells. In all these the ultimate character of the bodies is the same.

We come now to a subject which has been much discussed since the appearance of the "Cellular Pathologie." No pathologist before, since the time of Cruveilhier, who was the first to accurately describe the coagula found in blood-vessels, has found a satisfactory account of their origin and history. This Virchow does, in his doctrine of thrombosis, the process by which a coagulum of blood is formed within the vessel. According to Virchow, soon after the formation of these *thrombi*, their fibrin breaks down and becomes granular. The corpuscles lose their coloring matter, become crenated, and afterwards almost entirely disappear. A yellow-white, puriform liquid appears, in which the partially altered white corpuscles remain.

The fluid is not pus, although it appears so superficially; nor is the process suppurative, since it occurs in vessels in which no signs of inflammatory action are found. Thrombi may coexist with phlebitis; or, on the other hand, inflammation of the vascular walls "may unquestionably exist when the current of blood within the vessels of the affected part is perfectly free and unobstructed." But according to Virchow, in arteritis or phlebitis, the morbid products are formed in the walls of the vessel, and not in its calibre.

The thrombus is frequently confined to the seat of its origin extending as far as in the next large vessel, into which it empties; but frequently the deposition of layers of coagulum may prolong it beyond the mouth of the branch into the trunk, in the same direction with the current of the blood. Now portions of this may be detached by the current and carried into the general circulation, constituting emboli. If thus set free it will be carried along the venous channels to the heart, and thence proceeding in the pulmonary artery, will be detained in one of its branches. This detention will of course exclude the supply of blood to the tissues fed by the vessels.

It is from these data that Virchow accounts for these metastatic abscesses, which have been generally thought to dnnote pyemia.

"If," he says, "ulceration takes place in one of the valves of the heart, not by means of the formation of pus, but in consequence of an acute or chronic softening, crumbling fragments of the surface of the valve are borne away by the stream of blood, and reach with it far distant points, as the spleen, the kidneys, etc."

It is of course denied by Virchow that such metastases originate in the introduction and conveyance of pus in the blood. He denies its absorption; but he admits that corrupted juices may enter the blood, and lay hold upon certain points, for which the former has a special affinity. (See previous page.)

It is supposed by Virchow that in this latter way another class of metastases besides those attributable to emboli are caused. To this class belongs that metastatic pleurisy which develops itself without any metastatic abscess in the lungs and that seemingly rheumatic articular affection in which no distinct deposit is found in the joints; and further, that diffuse gangrenous inflammation of the subcutaneous tissue which cannot well be accounted for, unless we suppose a more chemical mode of infection.

Besides the foregoing, the volume entitled "*Cellular Pathologie*" gives the intimate anatomy and the physiological and pathological relations of the nervous system, and a classification of other normal tissues; the subject of nutrition; fatty, amyloid, and other forms of degeneration; while a large portion of it is occupied with an account of Virchow's observations of the process and transformations in growth of a normal kind, and these should be understood in order to estimate the value of the pathological doctrines. An analysis of these must be reserved for a future occasion, and opportunity recurring, we shall do our best towards a *résumé* of Virchow's observations in these departments, reserving until then any intimation of dissent. The doctrines can be invalidated only by deciding the incorrectness of the data upon which they are founded, and this is the province of the microscopist. If the data be true, the dissidence which has from the earliest dawn of medicine prevailed with regard to every feature of disease must disappear, and empiricism in pathology give place to the previsions and principles of science. We are ourselves in great measure, like Virchow's early auditors, convinced.

Comment upon the doctrines is valueless except by those who have an equal degree, if not extent, of proficiency with Virchow in pathological observations. We accord our conviction, that at no time has a volume in medicine with scientific pretensions at all to be compared to those of the "Cellular Pathologie" appeared. Nothing can exceed the easy mastery Virchow has shown over the details of all the subject, nor the evidence of ability to cope in a truly scientific manner with the great problems everywhere encountered on the field of pathology. At every step he has transcended the *rôle* of the mere observer with his barren accumulation of fact, and has given us their *rationale.* Few observers, especially in the world of medicine, ever seem to have had any conception that nature furnishes us with the facts or phenomena alone, and not the *philosophy* of them; *that* must be developed in the mind of the observer; the only difficulty in the case being, that the philosophy must be *that* of the facts, and not a philosophy or speculation discordant with them.

This service Virchow has competently striven to render to the scientific student, and he will probably stand hereafter as the first author in the long list of periods who has succeeded in doing in anything like the same degree or the same extent —a *scientific* work in pathology. He has signalized his difference in these respects from all other observers in the most fundamental way; passing, by dint of nice critical acumen, beyond the mere fallacious interpretation which so readily springs to the natural mind on the observation of facts, to that relationship between them which, once detected, furnishes the needed criterion of the truth or falsehood of the significance we at first sight assign to them.

Medicine, no less than pathology, is replete with such fallacies, and its professors must learn that philosophy as well as observation, intellectual resources as well as industry, are equally needed. Indeed, it is no more true that philosophy is at fault in medicine than it has been in natural history or mathematics. All the sciences equally receive their gains not from the mere collator of facts, but from the student of intellectual resources, who comes in the order of the method of discovery and investigation after the other, and employs his time and capacities in *using* the facts brought by the former. Whence now does he acquire his philosophy? Certainly not from the facts or phenomena given by nature, for these are solely the *subject* of his intellectual activity or life, but from some interior source, internal to himself, and not furnished by any fact of nature. Of course, as we just said, his philosophy must involve the facts, and consequently presupposes them; but it *supposes* also an evolution of powers which belongs to him, and constitutes the difference between him and the mere collator of facts, whose apprehension of them may be proved, on analysis, to be a confusion, and whose bungling attempts at elucidation will be a blunder. Considered in this view as to the rank and importance of his work, we reaffirm our starting sentence, that Virchow has opened a new era in the science of pathology; and whatever modifications or corrections his work may undergo, they can be made in no other way than on the principles and by the methods he has himself so ably employed.

It is evident to us that the cellular pathology has given a new impulse to these investigations, and that it will induce nothing less than a very thorough revision of the present accounts of the histology of the animal tissues, and that in this work Virchow himself may be expected to go further than he has yet attained in the results of micrology. A single casual observation, induced by his account of the connective tissue of the body, [illegible] led to some results which, with improved optical means and appliances at our command, we hope to verify. Sufficient is known to justify the belief that the genesis of muscular fibrillæ will be found to be allied, with some modifications, to the formation of the connective tissue corpuscle.—*Am. Med. Monthly.*

